# Fast Convolution Filter-Bank Based Non-Orthogonal Multiplexed Cognitive Radio (NOMCR) Receiver Design Using Cyclostationarity Based FRESH Filtering

**DOI:** 10.3390/s18061930

**Published:** 2018-06-13

**Authors:** Jayanta Datta, Hsin-Piao Lin

**Affiliations:** 1 Department of Electrical Engineering and Computer Science, National Taipei University of Technology , Daan District, Taipei City 10608, Taiwan; 2 Department of Electronic Engineering, National Taipei University of Technology, Daan District, Taipei City 10608, Taiwan; hplin@ntut.edu.tw

**Keywords:** multicarrier, NOMCR, cyclostationarity, ANN

## Abstract

Non-orthogonal multiple access (NOMA) systems are being considered as candidates for 5G wireless systems due to their promise of improved spectral efficiency. NOMA schemes are being combined with popular multicarrier schemes such as orthogonal frequency division multiplexing (OFDM) to take advantage of the benefits of multicarrier signals. A variant of the power domain NOMA is Layer Division Multiplexing (LDM). The most commonly deployed power domain LDM scheme involves successive interference cancellation (SIC) based decoding at the receiver. Fast convolution based filtered-OFDM (FC-F-OFDM) systems are becoming popular among 5G wireless access technologies due to their ability to process 5G physical layer signals efficiently. In this work, firstly, a cognitive multicarrier non-orthogonal multiplexed system based on the concept of LDM is discussed, which uses FC-F-OFDM and conventional OFDM as its component layers. Secondly, cyclostationary FREquency SHift (FRESH) filter based SIC decoding is used at the receiver side, which also utilizes artificial neural network (ANN) processing. Computer simulations indicate that the system provides good bit error rate (BER) performance under frequency selective Rayleigh fading channels.

## 1. Introduction

Non-orthogonal multiple access (NOMA) is currently being considered as a promising wireless multiple access technology capable of meeting the demands of 5G wireless systems. The fundamental idea of NOMA is multi-user spectrum sharing within a resource block through power-domain multiplexing [1]. This is different from orthogonal multiple access (OMA) schemes which rely on time-domain/frequency domain multiplexing. In comparison to orthogonal multiple access waveforms, non-orthogonal multiple access (NOMA) waveforms offer a set of desirable potential advantages such as enhanced spectrum efficiency, higher connectivity and reduced latency with high reliability. A slight variant of the power domain NOMA concept is the so-called Layer Division Multiplexing (LDM) technology for achieving a high data rate in the cloud transmission network [2,3,4]. Similar to conventional NOMA, in LDM, layers of signals with different power levels are superposed on each other, each layer delivering a different service. From an information-theoretic point of view, the approach is referred to as superposition coding (SC) [5]. An LDM system can comprise of 2 layers where the upper layer with higher power allocation may deliver mobile services and the lower layer may be used to serve high data rates such as HDTV [2,3,4]. The LDM concept has been standardized for the next generation American Digital Television standard ATSC 3.0 and has been initiated for the 3GPP project under the then name Multi-User Superposition Transmission (MUST) [6,7]. Apart from power-domain multiplexed NOMA or LDM, multi-user spectrum sharing can also be achieved through cognitive radio (CR) technology, where the secondary users (SU) can either opportunistically or collaboratively access the spectral bands occupied by the licensed user/primary user (PU) of that band. The primary objective of both NOMA and CR technology is efficient spectrum utilization. However, the enhancement of spectrum efficiency is achieved by these two closely related wireless technologies from two different perspectives. While CR facilitates the low-priority secondary access either in an opportunistic manner or collaborative manner, NOMA technology enables the simultaneous transmission of multiple users’ waveforms by allocating different power levels. A clever combination of CR and NOMA, referred to as the cognitive NOMA, can lead to an improved spectrum sharing approach to further enhance spectrum utilization. Some of the advantages offered by the cognitive NOMA approach are improved spectrum efficiency, massive connectivity, low latency and greater fairness [6,7,8]. Moreover, by adopting a multicarrier waveform framework, the spectrum utilization of cognitive NOMA can be further improved in a fragmented dynamic spectrum access (DSA) scenario. Orthogonal Frequency Division Multiple Access (OFDMA) is the most popular multicarrier signaling scheme. However, owing to its use of rectangular pulse shaping filters, the conventional OFDMA suffers from high spectral side-lobes, which leads to undesirable adjacent channel interference (ACI). Hence, conventional OFDM based cognitive NOMA can lead to harmful interference to the primary users of the spectrum. Hence, an alternative adaptive and flexible physical layer waveform with high spectral efficiency is required to achieve interference avoidance and optimized spectrum utilization in a densely deployed 5G network. Filtered multicarrier waveforms with fast convolution-based processing are promising candidates for this purpose [9,10]. The idea of a fast convolution-based filter-bank (FC-FB) is that the convolution of a long data sequence with a pulse shaping filter response can be achieved over frequency domain data blocks with overlapped block processing. Each sub-band can be easily configured for unequal bandwidths, different center frequencies and adjustable sample rate conversion factors [9,10]. Owing to their design flexibility and enhanced spectrum utilization, the FC-FB based waveform architecture is now being considered as a potential candidate for future 5G wireless technology. In comparison to conventional orthogonal multicarrier waveform like OFDM, FC-FB based multicarrier systems offer higher design flexibility as well as superior peak-to-average power ratio (PAPR) reduction performance, in addition to higher spectral efficiency. Hence, FC-FB based multicarrier waveforms seem to be perfect candidates for waveform architecture in cognitive NOMA systems.

The advantage of using FC-FB by the secondary users (SU) is that the filter-bank structure allows flexible sub-division of the available frequency band into sub-bands, each of which may be scanned by the SU transmitter for evaluating the PU interference temperature (IT) threshold [11]. Based on the IT threshold, the SU transmitter can apply power control and design a fast convolution based sub-band filtered waveform [10], in which the SU transmissions are superimposed on those sub-bands with the PU transmission where the IT threshold is not exceeded. This is an example of a non-orthogonal cognitive underlay system, where the SU system is under-laid with the PU system. Compared to an underlay OMA network, more efficient spectrum utilization can be achieved by applying the NOMA protocol in CR scenario. This is attributed to the fact that unlike OMA-based underlay, a greater number of SUs in the underlay NOMA system can simultaneously receive individual signals in the same shared spectrum, thereby improving SU connectivity as well as throughput for SU access. This kind of cognitive NOMA concept has been presented in References [6,12], which report the outage probability as well as diversity analysis in a large-scale underlay cognitive NOMA network. Moreover, the idea of cognitive NOMA has also been extended to a cooperative relay scenario, as reported in References [6,13]. However, the application of FC-FB waveform architecture has not been reported in these works. 

Despite the enormous possible advantages offered by the FC-FB based cognitive NOMA system, interference management remains a critical issue, due to the fact that such systems are highly interference limited. These systems suffer from inter-network interference caused by power domain multiplexing of the SU and PU transmissions. Apart from transmit side power control, the SU receiver needs to implement inter-network interference cancellation strategies to mitigate interference arising from PU transmission. While most works on non-cognitive as well as cognitive NOMA are primarily focused on transmitter side power control [1,2,3,4,6,7,12,13], few have explored the issue of enhanced signal detection at the SU receiver under the influence of strong PU interference. In most existing works, the receiver side processing in power-domain NOMA systems primarily involves conventional successive interference cancellation (SIC) based multi-user detection. However, such a scheme only relies on the allocated power levels of the NOMA signal components. It does not exploit the waveform structure for signal detection. 

Most multicarrier waveforms exhibit periodicities with their second order statistics (SOS). These periodicities result in specific correlation patterns in the spectral properties of the signals, which may be detectable even at low signal-to-noise ratios (SNRs) [14,15]. These specific periodicity patterns are referred to as cyclostationary signatures [16]. User specific cyclostationary signatures can be induced in the FC-FB based SU-NOMA waveform at the transmitter side. A simple example is the cyclic prefix (CP) in conventional multicarrier systems. Spectral Correlation Density (SCD) function can be computed at the receiver side and artificial neural network (ANN) [17,18] can be applied to detect the embedded cyclostationary signature for NOMA system recognition. The combination of cyclostationarity and ANN can be extended to implement an adaptive frequency shift (FRESH) [19,20,21,22] filtering at the receiver to extract the signal of interest (SOI) through successive interference cancellation (SIC). Most multicarrier wireless signals are appended with a cyclic prefix (CP) to combat the inter-symbol interference (ISI) caused by frequency selective fading channel. It is assumed that the CP length used by the PU-OFDM is different from that used by the SU-NOMA FC-FB system. Hence the FRESH filter can take advantage of the cyclostationarity induced by the CP of PU-OFDM to extract the PU-OFDM signal first. The extracted PU-OFDMA signal can then be subtracted from the received signal mixture, yielding an initial estimate of the FC-FB based SU-NOMA. Filter-bank demodulation can be performed on the initial estimate to recover the CP-NOMA blocks corrupted by channel noise. The signal can be subject to a second FRESH filter which can extract the desired SU-NOMA signal. Such receiver side neuromorphic processing can prove beneficial in cognitive NOMA scenarios under very low SNR conditions, where multiple PU and SU units attempt to coexist together. Due to the non-Gaussian nature of the residual signal, the ANN based cyclic FRESH receiver structure can prove beneficial in estimating the NOMCR individual signal components. 

### 1.1. Contributions

The contributions of this paper are to:

- Design a fast convolution filter-bank (FC-FB) based non-orthogonally multiplexed cognitive radio (NOMCR) system.
- Devise a neuromorphic receiver structure which exploits the spectral redundancy features in the superimposed PU and SU signals to jointly equalize the channel and extract the desired SU signal under interference limited non-orthogonal spectrum sharing scenarios.

The paper focusses on communication in a cyclostationary interference limited spectrum sharing environment, where the PU and SU transmissions are non-orthogonally superimposed. In this work, the cyclostationarity characteristics of the superimposed PU and SU signals are taken into account, as well as the non-linear prediction capability of the de-noising neural network. These two features are utilized to design a neuromorphic FRESH receiver, which is a combination of an adaptive cyclostationary interference canceller and a channel equalizer. The proposed technique is capable of achieving good BER performance as well as better signal-to-interference and noise-ratio (SINR) compared to the conventional recursive least squares (RLS) based FRESH receiver. 

### 1.2. Organization

Section 2 provides a description of the cognitive NOMCR system, with each of its sub-sections outlining the conventional OFDM-based LDM scheme and proposed fast convolution processing based NOMCR system. The outline of the fast convolution based filtered OFDM transceiver model can be obtained from Section 2.2.1, while Section 2.2.2 provides details of the system model of the proposed fast convolution based NOMCR system. Section 3 provides details about the optimal FRESH as well as RLS based blind adaptive FRESH receiver. Section 4 presents the proposed neuromorphic FRESH receiver structure. Section 4.1 and Section 4.2 describe the motivation and procedure of implementing the proposed receiver. Section 5 presents the computer simulation results. Section 6 provides possible future applications of the proposed receiver. Section 7 concludes the paper with a general discussion.

## 2. Non-Orthogonally Multiplexed Cognitive Radio (NOMCR) System

### 2.1. Conventional OFDM-LDM

Cloud transmission network (Cloud-Txn) with layer division multiplexing (LDM) is a non-orthogonal multiplexing transmission technique which provides a very high data rate [2,3,4]. Conventional multicarrier LDM adopts a technique similar to OFDM based power domain NOMA. In such a scheme, waveforms corresponding to multiple services such as digital broadcasting or multicasting are transmitted in different signal layers within a single radio frequency (RF) channel. Contrary to traditional time division multiplexing (TDM) or frequency division multiplexing (FDM) systems, each transmission layer in LDM occupies the full frequency spectrum and full-time duration. As evident, the LDM scheme provides full frequency diversity and time diversity to the waveforms in all the layers of the system. For a 2-layer LDM, the upper layer (UL) is responsible for delivering service to mobile users under low SNR and harsh multipath conditions. Hence it is designed to possess high transmission power and use very strong coding and digital modulation schemes such as ¼-low density parity check code (LDPC) with QPSK. On the other hand, the lower layer (LL) is responsible for providing high data rate service to fixed users which may use large directional antennas to provide high antenna gain. Hence the LL is designed to use lower transmission power, weaker channel coding and larger signal constellation sizes, for e.g., 64-QAM, 256-QAM etc. Details of the OFDM based LDM transceiver can be obtained from Reference [4]. The traditional LDM concept can be extended to the cognitive radio (CR) scenario. In a CR system based on interference temperature (IT) constraint [11], the secondary user (SU) can allocate its data on those portions of the frequency bands where the tolerable IT is not exceeded. So, in those sub-bands, the SU can superimpose its modulated data streams on the primary user (PU) data. In essence, this is a non-orthogonal multiplexed cognitive radio (NOMCR) transmission scheme where the SU transmission is opportunistically superposed on the PU data in a non-orthogonal fashion. This kind of non-orthogonal cognitive multicarrier system can lead to higher spectral efficiency. However, due to the use of rectangular pulse shaping filters, the out-of-band (OOB) radiation in conventional OFDM based systems is high leading to harmful adjacent channel interference (ACI) to the PU transmissions. Hence there is need to use alternative multicarrier processing schemes for the NOMCR transmissions.

### 2.2. Fast Convolution Filtered OFDM Based-NOMCR System

Fast convolution processing based filtered multicarrier systems can provide enhanced data rates as well as lower out-of-band (OOB) radiation, the latter being attributed to the use of suitable pulse shaping filters. Fast convolution filtered OFDM (FC-F-OFDM) seems to be an attractive choice for the design of NOMCR system. Hence in this work, we propose to apply fast convolution-based filter-bank processing instead of the conventional OFDM modulation. 

In FC-F-OFDM transmitter, multiple narrow-band low-rate signals denoted by **x**^su^_m_, where m = 0, 1, …, M − 1, are filtered by pulse shaping filters with adjustable frequency responses and adjustable sampling rates. The filtered signals are then multiplexed into a single wideband signal, denoted by **s**^su^. This is referred to as a synthesis filter bank (SFB). At the receiver side, the dual operation called analysis filter bank (AFB) takes place. In AFB, the incoming high rate wideband signal is split into multiple narrow-band signals and reverse operations to the transmit side processing take place to recover the transmitted data. Details of the transmitter and receiver side processing operations in FC-F-OFDM system can be obtained from References [9,10]. In this section, the mathematical equations describing the FC-FB transmitter and receiver are mentioned briefly. 

#### 2.2.1. Fast Convolution Filtered OFDM (FC-F-OFDM) Transceiver

Let N^su^_data_ be the number of subcarriers in the SU- FC-F-OFDM symbol and let N^su^_cp_ be its cyclic prefix. Then, the length of the SU- FC-F-OFDM symbol in time samples is given by, N^su^_sym_ = N^su^_data_ + N^su^_cp_. At the transmitter side, FC-F-OFDM synthesis transform operates on the CP-OFDM pre-processed SU data on a sub-band basis to generate the final wide-band signal. The high rate mth sub-band waveform, **w**^su^_m_, of the FC-F-OFDM based SU system can be generated as

(1) $$w_{m}^{\mathrm{su}}=F_{m}^{\mathrm{su}}\cdot x_{m}^{\mathrm{su}}=\left[\mathrm{diag}\left(F_{m,0}^{\mathrm{su}},F_{m,1}^{\mathrm{su}},\ldots ,\;F_{m,R_{m}-1}^{\mathrm{su}}\right)\right]\cdot x_{m}^{\mathrm{su}}$$

where **F**^su^_m_ is the block diagonal SFB transform matrix on the narrow-band, low-rate mth sub-band signal **x**^su^_m_ and R_m_ is the total number of sub-blocks of **x**^su^_m_. The SFB operations of the rth sub-block of **x**^su^_m_ are expressed mathematically as

(2) $$F_{m,r}^{\mathrm{su}}=S_{N_{\mathrm{sym}}^{\mathrm{su}}}^{\mathrm{su}}\cdot {\left(W_{N_{\mathrm{sym}}^{\mathrm{su}}}^{\mathrm{su}}\right)}^{-1}\cdot M_{m,r}^{\mathrm{su}}\cdot D_{m}^{\mathrm{su}}\cdot {\left(p_{L_{m}}^{\mathrm{su}}\right)}^{\left(L_{m}/2\right)}\cdot W_{L_{m}}^{\mathrm{su}}$$

where $W_{L_{m}}^{\mathrm{su}}$ is the L_m_-by-L_m_ DFT matrix, ${\left(W_{N_{\mathrm{sym}}^{\mathrm{su}}}^{\mathrm{su}}\right)}^{-1}$ is the $N_{\mathrm{sym}}^{\mathrm{su}}$-by-$N_{\mathrm{sym}}^{\mathrm{su}}$ IDFT matrix, ${\left(p_{L_{m}}^{\mathrm{su}}\right)}^{\left(L_{m}/2\right)}$ is the circulant permutation matrix which cyclically left shifts L_m_-by-L_m_ identity matrix by L_m_/2 positions, $M_{m,r}^{\mathrm{su}}$ is the $N_{\mathrm{sym}}^{\mathrm{su}}$-by-L_m_ frequency domain mapping matrix which maps the L_m_ incoming samples of the sub-block to the respective center frequency in the sub-band and $D_{m}^{\mathrm{su}}$ is the L_m_-by-L_m_ diagonal matrix with frequency domain window weights of the mth sub-band on its main diagonal. 

The frequency domain diagonal matrix of filter coefficients can be expressed as

(3) $$D_{m}^{\mathrm{su}}=\mathrm{diag}\left(\left[\begin{array}{c} 0_{\left(\lceil \frac{\left[L_{m}-L_{\mathrm{act},m}\right]}{2}\rceil -L_{\mathrm{TBW},m}\right)-\mathrm{by}-1}^{\mathrm{su}}\\ d_{0,m}^{\mathrm{su}}\\ \ldots \\ d_{L_{\mathrm{TBW}}-1,m}^{\mathrm{su}}\\ 1_{L_{\mathrm{ACT},m}-\mathrm{by}-1}\\ d_{L_{\mathrm{TBW}}-1,m}^{\mathrm{su}}\\ \ldots \\ d_{0,m}^{\mathrm{su}}\\ 0_{\left(\lfloor \frac{\left[L_{m}-L_{\mathrm{act},m}\right]}{2}\rfloor -L_{\mathrm{TBW},m}\right)-\mathrm{by}-1}^{\mathrm{su}} \end{array}\right]\right)$$

where $L_{\mathrm{ACT},m}$ denotes the number of active subcarriers for the mth sub-band of the SU- FC-F-OFDM, $L_{\mathrm{TBW},m}$ defines the transition bandwidth (TBW) of mth sub-band and $d_{i,m}^{\mathrm{su}}$, i = 0, 1, …, $L_{\mathrm{TBW}}-1$, are the non-trivial frequency domain filter weights in the transition band. The mth sub-band pass-band weights are represented by the $L_{\mathrm{ACT},m}$-by-1 column vector of ones, denoted by boldface $1_{L_{\mathrm{ACT},m}-\mathrm{by}-1}$. On the other hand, stop-band weights of the mth sub-band are denoted by the $\left(\lfloor \frac{\left[L_{m}-L_{\mathrm{act},m}\right]}{2}\rfloor -L_{\mathrm{TBW},m}\right)$-by-1 column vector of zeros, denoted by the boldface $0_{\left(\lfloor \frac{\left[L_{m}-L_{\mathrm{act},m}\right]}{2}\rfloor -L_{\mathrm{TBW},m}\right)-\mathrm{by}-1}^{\mathrm{su}}$. 

The receiver side AFB processing operations for the rth sub-block of the mth sub-band can be expressed as

(4) $$G_{m,r}^{\mathrm{su}}=S_{L_{m}}^{\mathrm{su}}\cdot {\left(W_{L_{m}}^{\mathrm{su}}\right)}^{-1}\cdot {\left(P_{N_{\mathrm{sym}}^{\mathrm{su}}}^{\mathrm{su}}\right)}^{\frac{N_{\mathrm{sym}}^{\mathrm{su}}}{2}}\cdot D_{m}^{\mathrm{su}}\cdot {\left(M_{m,r}^{\mathrm{su}}\right)}^{T}\cdot W_{N_{\mathrm{sym}}^{\mathrm{su}}}^{\mathrm{su}}$$

The receiver side sub-band processing operation, $G_{m,r}^{\mathrm{su}}$, acts as a matched filter on the received signal to recover the CP-OFDM for the mth sub-band. As evident from the above discussion, different sub-bands can have a different number of sub-carriers.

#### 2.2.2. FC-F-OFDM Based NOMCR System Model

Let N^pu^_data_ be the number of subcarriers in the PU-OFDM symbol and N^pu^_cp_ be its cyclic prefix. Then, the length of the PU-OFDM symbol in time samples is given by, N^pu^_sym_ = N^pu^_data_ + N^pu^_cp_. The transmitter side baseband PU-OFDM signal in time domain can be represented by the following mathematical equation

(5) $$s^{\mathrm{pu}}\left(n\right)=\left(\frac{1}{\sqrt{N_{\mathrm{data}}^{\mathrm{pu}}}}\right)\sum_{m=-\infty }^{\infty }\sum_{k=0}^{N_{\mathrm{data}}^{\mathrm{pu}}-1}b_{m,k}^{\mathrm{pu}}\cdot p\left(n-m\cdot N_{\mathrm{sym}}^{\mathrm{pu}}\right)\cdot e^{j\cdot 2\pi k\cdot \frac{n-m\cdot N_{\mathrm{sym}}^{\mathrm{pu}}}{N_{\mathrm{data}}^{\mathrm{pu}}}},\;n=0,1,\ldots ,\;N_{\mathrm{sym}}^{\mathrm{pu}}-1,$$

where **p**(n) is the transmitter side pulse shaping filter, **b**^pu^_m,k_ is the data symbol at the kth subcarrier of the mth OFDM symbol and **s**^pu^(n) is the transmit side PU-OFDM signal. In matrix format, the PU-OFDM signal can be expressed mathematically as

(6) $$s^{\mathrm{pu}}\left(n\right)=F_{\mathrm{cp}}^{\mathrm{pu}}\cdot W^{H}\cdot P\cdot b^{\mathrm{pu}},\;\mathrm{where}\;F_{\mathrm{cp}}^{\mathrm{pu}}=\left[\begin{array}{cc} 0 & I_{N_{\mathrm{cp}}^{\mathrm{pu}}}\\ I_{N_{\mathrm{data}}^{\mathrm{pu}}-N_{\mathrm{cp}}^{\mathrm{pu}}} & 0\\ 0 & I_{N_{\mathrm{cp}}^{\mathrm{pu}}} \end{array}\right]$$

where **F**^pu^_cp_ is the cyclic prefix (CP) matrix, **W** is the DFT matrix, **P** is the diagonal matrix of frequency domain pulse shaping filter coefficients and **b**^pu^ is the column vector of N^pu^_data_-by-1 PU data samples. 

As evident from the formulation of the PU-OFDM signal in Equations (5) and (6), the total number of SU data samples, N^su^_data_, within each SU sub-band is a subset of the total number of PU-OFDM data samples within that band. If there are M total number of sub-bands belonging to the SU, then the FC-F-OFDM based SU signal component for the entire band can be mathematically represented by

(7) $$w_{m,\mathrm{LL}}^{\mathrm{su}}\left(n\right)=F_{m}^{\mathrm{su}}\cdot (g\cdot x_{m}^{\mathrm{su}}\left(n\right))$$

In Equation (7), the term ‘g’ represents the power level of the SU signal relative to the PU-OFDM signal. Let the total transmission power of the NOMCR signal be P_s_. The injection level for the FC-F-OFDM based SU signal is denoted by ρ dB, where $\rho =-20\times \log_{10}g$. As evident from Equation (7), to decode the SU signal, the receiver is required to first cancel the estimate of the PU-OFDM signal from the received NOMCR signal. Then the residual SU signal undergoes matched filtering per sub-band (i.e., inverse of the transmit side overlap-and-save fast convolution based digital filtering) to recover the sub-band CP-OFDM signal. 

**Received NOMCR Signal Model:**

Let $r_{\mathrm{NOMCR}}\left(n\right)$ be the received NOMCR signal, corrupted by AWGN **w** (n). Then the receiver side signal model can be expressed by,

(8) $$r_{\mathrm{NOMCR}}\left(n\right)=h_{\mathrm{pu}}⊗s^{\mathrm{pu}}\left(n\right)+h_{\mathrm{su}}w_{m,\mathrm{LL}}^{\mathrm{su}}\left(n\right)+w\left(n\right),$$

In (8), $h_{\mathrm{pu}}$ and $h_{\mathrm{su}}$ represent frequency selective channels corresponding to PU and SU transmissions respectively. Let the estimated PU-OFDM signal and its estimated channel be denoted by **ŝ**^pu^(n) and **ĥ**_pu_ respectively. Then the estimated high-power PU-OFDM signal can be treated as interference and cancelled from the received LDM signal. To obtain a reliable estimate of the higher power PU-OFDM signal, the received signal needs to undergo OFDM demodulation, channel estimation and equalization, de-interleaving, and channel decoding to obtain a reliable bit decision estimate for the PU-OFDM. The estimated bit sequence is re-encoded, re-interleaved and re-modulated to generate the reconstructed transmitted PU-OFDM signal. Although the reconstruction process incurs higher complexity, it provides a reliable estimate. 

**SU Signal Model with PU interference estimation and cancellation:**

The estimate of the SU signal, corrupted by the wireless fading channel, can be obtained as

(9) $$\begin{array}{c} r_{\mathrm{su}}\left(n\right)=h_{\mathrm{su}}⊗w_{m,\mathrm{LL}}^{\mathrm{su}}\left(n\right)+\left(h_{\mathrm{pu}}⊗s^{\mathrm{pu}}\left(n\right)-{\hat{h}}_{\mathrm{pu}}⊗{\hat{s}}^{\mathrm{pu}}\left(n\right)\right)+w\left(n\right)\\ =h_{\mathrm{su}}⊗w_{m,\mathrm{LL}}^{\mathrm{su}}\left(n\right)+i^{\mathrm{pu}}\left(n\right)+w\left(n\right) \end{array}$$

In (9), $i^{\mathrm{pu}}\left(n\right)$ denotes the residual interference after estimated PU-OFDM signal cancellation. The channel equalization of $r_{\mathrm{su}}\left(n\right)$ can be performed with the help of the channelization weights of the fast convolution filter-bank structure. In other words, the sub-block based equalization is embedded within the matched filter. The frequency domain weights of the embedded $R_{m}$th sub-block equalizer of the mth sub-band can be calculated by

(10) c^Rm,n=(H^Rm,n∗·cRm,n∗g)|H^Rm,n·cRm,n|2+|H^Rm,ń·cRm,ń|2+(1δ)

where ${\hat{H}}_{R_{m},n}$ denotes the estimated $R_{m}$th sub-block frequency response at the nth FFT bin and $c_{R_{m},n}$ denotes the RRC pulse shaping filter coefficients of the $R_{m}$th sub-block, nth FFT bin. The channel estimation can be performed by any standard channel estimation method like the scattered pilot-based channel estimation [23,24]. The demodulation of each sub-block is carried out by the matched filter

(11) $${\hat{G}}_{m,r}^{\mathrm{su}}=S_{L_{m}}^{\mathrm{su}}\cdot {\left(W_{L_{m}}^{\mathrm{su}}\right)}^{-1}\cdot {\left(P_{N_{\mathrm{sym}}^{\mathrm{su}}}^{\mathrm{su}}\right)}^{\frac{N_{\mathrm{sym}}^{\mathrm{su}}}{2}}\cdot C_{m}^{\mathrm{su}}\cdot {\left(M_{m,r}^{\mathrm{su}}\right)}^{T}\cdot W_{N_{\mathrm{sym}}^{\mathrm{su}}}^{\mathrm{su}}$$

The RHS of (11) is almost similar to (4) except for the embedded sub-block equalizer matrix, $C_{m}^{\mathrm{su}}$, containing the terms ${\hat{c}}_{R_{m},n}$ along its main diagonal and zeros elsewhere. The demodulated SU signal can be expressed by

(12) $$x_{m,r}^{\mathrm{SU}}={\hat{G}}_{m,r}^{\mathrm{su}}\cdot r_{\mathrm{su}}\left(n\right),\;\mathrm{where}\;r\;=\;1,\;2,\;\ldots ,\;R_{m}$$

(13) $$x_{m,\mathrm{est}}^{\mathrm{SU}}={\left[{\left(x_{m,1}^{\mathrm{SU}}\right)}^{T}\;{\left(x_{m,2}^{\mathrm{SU}}\right)}^{T},\;\ldots ,\;{\left(x_{m,R_{m}}^{\mathrm{SU}}\right)}^{T}\right]}^{T}$$

The term, $x_{m,\mathrm{est}}^{\mathrm{SU}}$, on L.H.S of (13) denotes the estimate of the CP-OFDM signal transmitted by the SU on its mth sub-band. The remaining steps involve CP removal, OFDM demodulation (i.e., FFT), de-interleaving and channel decoding to recover the transmitted bit-sequence by the SU. As evident from Equation (9), there seems to be cross-layer interference (CLI) corrupting the NOMCR transmission. This leads to impaired bit error rate (BER) performance at the receiver. The problem can be overcome if we use a joint interference cancellation and equalization strategy at the NOMCR receiver. This strategy makes use of the cyclostationary properties of the 2 layers of multiplexed signals to separate the 2 layers of transmissions and decode them. In this work, we apply the principle of cyclostationary adaptive FRESH filtering to a neural network predictor [25,26,27] to extract the individual layers of signals and perform successive interference cancellation (SIC). Owing to the CP, the cyclostationary period of the PU-OFDM signal is given by the total length of the PU-OFDM symbol (i.e., CP length + IFFT size). Therefore, the CP induced cyclic frequency of the PU-OFDM signal is given by, $\alpha_{k}^{\mathrm{pu}}$ = kNsympu, where k = 0, 1, …, $N_{\mathrm{sym}}^{\mathrm{pu}}$ − 1. 

Moreover, as evident from the waveform structure of FC-F-OFDM, different sub-bands can have different number of sub-carriers. This means the CP sizes of the sub-bands can be different. Due to different CP lengths, the CP induced cyclostationary periods of each sub-band of the FC-FB will be different from that of the PU-OFDM. Moreover, even if the numbers of samples in each sub-band are equal, the CP length can be adjusted to be different from that of the PU-OFDM. If $\alpha_{k,m}^{\mathrm{su}}$ denotes the kth cyclic frequency of the mth sub-band of the SU-FC-FB, then $\alpha_{k,m}^{\mathrm{su}}$ = k(Nm,symsu), where the cyclostationary period of SU-FC-FB’s mth sub-band is denoted by $N_{m,\mathrm{sym}}^{\mathrm{su}}$. It is evident that the signal patterns of the individual components of the NOMCR signal can be utilized for implementing cyclic-FRESH filter based SIC receiver under a low SNR regime. 

## 3. Cyclostationarity

### 3.1. LCL-PTV Optimal FRESH Filtering

A complex-valued zero-mean signal is said to exhibit wide-sense (second-order) cyclostationarity (WSCS) with *cycle frequency* (CF) α ≠ 0 if and only if the cyclic autocorrelation function (CAF) denoted by the following mathematical equation [14,15,22]

(14) $$R_{xx}^{\alpha }=<E\left[x\left(t+\frac{\tau }{2}\right)x\cdot \left(t-\frac{\tau }{2}\right)\right]e^{-j2\pi \alpha t}=<R_{xx}\left(t,\tau \right)e^{-j2\pi \alpha t}>$$

exists and is not zero for some values of the lag parameter τ. In (14), <.> and E [.] denote infinite-time and ensemble average, respectively, and R_xx_(t,τ) is the statistical autocorrelation function of x(t). The discrete time domain CAF can be expressed by the following mathematical equation

(15) $$R_{\mathrm{xx}}^{\alpha_{k}}\left(l\right)=\left(\frac{1}{N_{0}}\right)\sum_{n=0}^{N_{0}-1}R_{\mathrm{xx}}\left(n,l\right)e^{-j2{\pi \alpha }_{k}n}$$

where α_k_ = k/N_0_, k = 0, 1, …, N_0_ − 1, are referred to as the cyclic frequencies and N_0_ is the cyclostationary period. The spectral correlation function (SCF), $S_{\mathrm{xx}}^{\alpha_{k}}\left(f\right)$, can be computed by the Fourier Transform of $R_{\mathrm{xx}}^{\alpha_{k}}\left(n\right)$ and can be expressed mathematically by

(16) $$S_{\mathrm{xx}}^{\alpha_{k}}\left(f\right)=\sum_{n=0}^{N_{0}-1}R_{\mathrm{xx}}^{\alpha_{k}}\left(n\right)e^{j2\pi \mathrm{fn}}$$

A FRESH filter is an optimal widely linear filter which exploits the spectral redundancy in the input signal to extract the desired cyclostationary signal. LCL-PTV filters belong to the class of FRESH filters, which exploit both the spectral redundancy and conjugate spectral redundancy to derive an estimate of the desired signal. The output signal $\hat{y}\left(n\right)$ for the input mixed signal r(n) (comprising desired signal y(n) and some unknown interference signal) is given by [19,20,21,22,28]

(17) $$\hat{y}\left(n\right)=\sum_{l=-\infty }^{\infty }h\left(n,l\right)\cdot r\left(l\right)+\sum_{m=-\infty }^{\infty }h^{c}\left(n,m\right)\cdot r^{*}\left(m\right)$$

The impulse responses of the FRESH filter h(n,l) and $h^{c}\left(n,l\right)$ can be expressed as

(18) $$\begin{array}{c} h\left(n,l\right)=\sum_{p}h_{p}\left(n-l\right)\cdot e^{j2{\pi \alpha }_{p}l}\\ h^{c}\left(n,l\right)=\sum_{q}h_{q}\left(n-l\right)\cdot e^{j2{\pi \alpha }_{q}^{c}l} \end{array}$$

Relationship between the input r(n) and estimated output $\hat{y}\left(n\right)$ of the FRESH filter is given by

(19) $$\hat{y}\left(n\right)=\sum_{l=-\infty }^{\infty }(\sum_{p}h_{p}\left(n-l\right)\cdot r_{p}\left(l\right)+\sum_{q}h_{q}\left(n-l\right)\cdot r_{q}^{c}\left(l\right))$$

where $r_{p}\left(n\right)$ = $r\left(n\right)\cdot e^{-j2{\pi \alpha }_{p}n}$, and $r_{q}^{c}\left(n\right)$ = $r^{*}\left(n\right)$·$e^{-j2{\pi \alpha }_{q}^{c}n}$. Based on (17)–(19), the system operation of LCL-FRESH filter can be represented by

(20) $$\begin{array}{c} \hat{y}\left(n\right)=\sum_{p}h_{p}\left(n\right)⊗\left[r\left(n\right)\cdot e^{j2{\pi \alpha }_{p}n}\right]+\sum_{q}h_{q}\left(n\right)⊗\left[r^{*}\left(n\right)\cdot e^{-j2{\pi \alpha }_{q}^{c}n}\right]\\ =\sum_{p}h_{p}\left(n\right)⊗r_{p}\left(n\right)+\sum_{q}h_{q}\left(n\right)⊗r_{q}^{c}\left(n\right) \end{array}$$

From Equation (22), it is evident that the LCL-FRESH filter can be regarded as two parallel FIR filter-bank systems. The 1st filter-bank and 2nd filter-bank are driven by the received signal and its conjugated version respectively. The outputs of the 2 filter-banks are added together to form the estimated version, $\hat{y}\left(n\right)$ of the desired SOI. The frequency shifts represented by $e^{j2{\pi \alpha }_{p}n}$ are used for aligning the spectral redundancies within $r_{p}\left(n\right)$, which are combined by the 1st filter-bank, {$h_{p}\left(n\right)$}. Similarly, the frequency shifts represented by $e^{-j2{\pi \alpha }_{q}^{c}n}$ are used for aligning the conjugate spectral redundancies within $r_{q}^{c}\left(n\right)$, which are combined by the 2nd filter-bank, {$h_{q}\left(n\right)$}. The optimal FRESH filter weights can be derived by minimizing the mean squared error (MSE) between the desired cyclostationary signal and its estimate. The derivation can be simplified if the MMSE is performed in the frequency domain. The frequency domain optimal FRESH filter equation can be expressed as [19,28]

(21) $$\hat{Y}\left(f\right)=\sum_{p}H^{\alpha_{p}}\left(f\right)\cdot R_{p}\left(f-\alpha_{p}\right)+\sum_{q}H^{\alpha_{q}}\left(f\right)\cdot R_{q}\left(-f+\alpha_{q}\right)$$

where $H^{\alpha_{p}}\left(f\right)=\mathrm{DFT}\left\{h_{p}\left(n\right)\right\}$, $H^{\alpha_{q}}\left(f\right)=\mathrm{DFT}\left\{h_{q}\left(n\right)\right\}$, $R_{p}\left(f\right)=\mathrm{DFT}\left\{r_{p}\left(n\right)\right\}$, $R_{p}\left(f\right)=\mathrm{DFT}\left\{r_{q}^{c}\left(n\right)\right\}$ and $\hat{Y}\left(f\right)=\mathrm{DFT}\left\{\hat{y}\left(n\right)\right\}$. The MSE between Y(f) and $\hat{Y}\left(f\right)$ can be expressed by

(22) $$\begin{array}{c} E_{\mathrm{mse}}\left(f\right)=E\left\{E\left(f\right)\cdot E^{*}\left(f\right)\right\},\;where\;E\;denotes\;expectation\;operator\\ =E\left\{\left(Y\left(f\right)-\hat{Y}\left(f\right)\right)\cdot {\left(Y\left(f\right)-\hat{Y}\left(f\right)\right)}^{*}\right\} \end{array}$$

The MSE can be obtained by taking its derivative with respect to the frequency domain filter weights $H^{\alpha_{p}}\left(f\right)$ and $H^{\alpha_{q}}\left(f\right)$, and setting equal to zero. According to the analysis in References [19,28], the MMSE solution to the optimum FRESH filter can be expressed as an optimum Wiener filter solution:

(23) $$H_{opt}=S_{RR}^{-1}\cdot S_{RY}$$

where $R=\left[DFT\left(\left[r_{1}\left(n\right),\;r_{2}\left(n\right)\ldots r_{p}\left(n\right),r_{1}^{c}\left(n\right),r_{2}^{c}\left(n\right),\ldots r_{Q}^{c}\left(n\right)\right]\right)\right]{}^{T}$, $S_{RR}=E\left\{R\cdot R^{H}\right\}$ and $S_{RY}=E\left\{R\cdot Y^{*}\right\}$. Hence from this formulation, the optimum FRESH filter coefficients corresponding to the PU-OFDM and each CP-OFDM based sub-band of the SU-FC-FB signals can be expressed as

(24) $$H_{opt}^{\mathrm{pu}}=S_{r_{nomcr}^{\mathrm{pu}}\cdot r_{nomcr}^{\mathrm{pu}}}^{-1}\cdot S_{r_{nomcr}^{\mathrm{pu}}.s^{\mathrm{pu}}}$$

(25) $$H_{opt}^{su,m}=S_{r_{nomcr}^{su,m}\cdot r_{nomcr}^{su,m}}^{-1}\cdot S_{r_{nomcr}^{su,m}.x_{m}^{su}}$$

where $r_{nomcr}^{\mathrm{pu}}$ and $r_{nomcr}^{su,m}$ denote the received NOMCR signal shifted by cyclic frequencies corresponding to the PU-OFDM and the mth sub-band of the SU-FC-FB signal, respectively. $s^{\mathrm{pu}}$(n) and $x_{m}^{\mathrm{su}}$(n) denote the PU-OFDM signal and the mth sub-band of the SU-FC-FB signal, respectively.

### 3.2. Conventional Blind Adaptive RLS-FRESH Filtering

**Figure 1:**
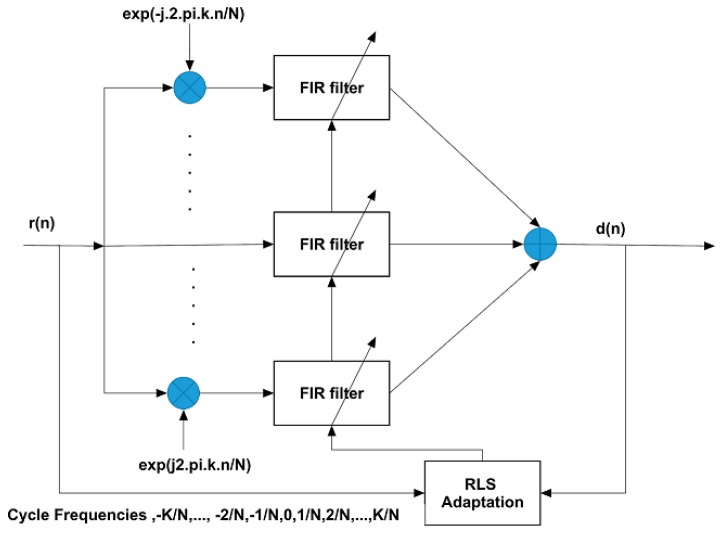
Basic Block Diagram of RLS based BA-LCL-FRESH Filter.

In (22), the impulse response coefficients of the optimal FRESH filter, $h_{p}\left(n\right)\}$, and $h_{q}\left(n\right)$, can be obtained either through direct calculation by solving the multivariate Wiener filtering problem [19] or by performing adaptive updating of the filter weights. For direct calculation of the optimal filter weights, the autocorrelation of the frequency shifted input signal vector and the cross-correlation between the input signal vector and the desired signal are required. However, practically it is unlikely to know these values a-priori. Hence an adaptive implementation of the FRESH filter is necessary. The basic structure of the BA-LCL-FRESH filter is shown below (Figure 1):

The steps involved in the Recursive Least Squares (RLS) based BA-LCL-FRESH filter in our NOMCR application can be tabulated as follows (Algorithm 1):
**Algorithm 1.** RLS Adaptation Algorithm Steps.Parameters: λ = forgetting factor, δ = initializing factor for P(0)Initialization:-       **P**(0) = $\delta^{-1}$·**I**, where **I** is the identity matrix of rank L       d(n) = r(n), i.e. received signal before cyclic frequency shift operation        $L=\sum_{p=1}^{P}L_{p}+\sum_{q=1}^{Q}M_{q}$ Computation:-        For n = 0, 1, 2, …              $r^{\alpha_{p}}\left(n\right)=\left[\begin{array}{c} r\left(n\right)e^{j2\pi \alpha_{p}n}\\ r\left(n-1\right)e^{j2\pi \alpha_{p}\left(n-1\right)}\\ \vdots \\ r\left(n-L_{p}+1\right)e^{j2\pi \alpha_{p}\left(n-L_{p}+1\right)} \end{array}\right]\;,\;p=1,2,\ldots ,P$            $r^{\beta_{q}}\left(n\right)=\left[\begin{array}{c} r^{*}\left(n\right)e^{j2\pi \alpha_{q}^{c}n}\\ r^{*}\left(n-1\right)e^{j2\pi \alpha_{q}^{c}\left(n-1\right)}\\ \vdots \\ r^{*}\left(n-M_{q}+1\right)e^{j2\pi \alpha_{q}^{c}\left(n-M_{q}+1\right)} \end{array}\right]\;,\;q=1,2,\ldots ,Q$                $r\left(n\right)=\;\left[\begin{array}{c} r^{\alpha_{1}}\left(n\right)\\ r^{\alpha_{2}}\left(n\right)\\ \vdots \\ r^{\alpha_{P}}\left(n\right)\\ r^{\alpha_{1}^{c}}\left(n\right)\\ r^{\alpha_{2}^{c}}\left(n\right)\\ \cdot \\ \cdot \\ r^{\alpha_{Q}^{c}}\left(n\right) \end{array}\right]$       $e\left(n\right)=d\left(n\right)-w^{H}\left(n-1\right)\cdot r\left(n\right)$: Compute the estimate of the a-priori error        $g\left(n\right)=\frac{P\left(n-1\right)\cdot r\left(n\right)}{\lambda +r^{H}\left(n\right)\cdot P\left(n-1\right)\cdot r\left(n\right)}$: Compute the gain vector        $P\left(n\right)=\lambda^{-1}\cdot P\left(n-1\right)-\lambda^{-1}\cdot g\left(n\right)\cdot r^{H}\left(n\right)\cdot P\left(n-1\right)$: Update the matrix P(n)       $w\left(n\right)=w\left(n-1\right)+e^{*}\left(n\right)\cdot g\left(n\right)$: Update the filter tap weight vector
Final Desired Signal Estimate: $\hat{y}\left(n\right)=w^{H}\cdot r\left(n\right)$

## 4. Adaptive Neuromorphic Joint FRESH Equalizer and Detector

### 4.1. Motivation

Artificial neural networks (ANN) are highly interconnected networks equipped with massive parallel processing functionality, which are inspired by the biological nervous system. Due to the massive parallelism and robustness offered by these neural networks, they are highly suitable for solving complex non-linear problems. In wireless communications applications such as channel equalization and automatic modulation classification (AMC), ANN-based receivers have been proven to outperform their conventional counterparts [26]. Due to their immense benefits, ANN is selected to perform the role of FRESH equalizer in this work. Basically, the ANN performs as an adaptive prediction filter, which can be used to predict the PU signal by utilizing the known cyclostationary features of the PU signal. The prediction noise is a combination of extracted SU-FC-FB signal and AWGN [26]. This means that the residual error signal of the cyclostationary prediction filtering is non-Gaussian in nature. Hence, use of conventional BA-RLS-FRESH joint equalization and data detection method will lead to performance degradation. However, ANN can perform the same role with much better performance. 

The primary objective of neural network training is to adaptively update the weights and biases to cause the output of the neural network predictor to approach the desired output signal. Evidently, this involves a certain amount of training data, which requires knowledge of a specific number of input-output pairs. If the past samples of the PU-OFDM signal are available, then they can be utilized as the desired output of the ANN based predictor to train the network. But from a practical standpoint, the exact PU-OFDM signal samples are not known in advance. Hence, in the NOMCR scenario, it is assumed that no prior information on the PU-OFDM is available except its cyclic frequencies, owing to its pilot arrangement and specific modulation format. Therefore, as an alternative to conventional ANN training, the received NOMCR signal can be used as the desired output signal to train the ANN. Owing to higher power allocation to the PU-OFDM signal and perfect knowledge of its cyclic frequencies at the SU receiver, the ANN can predict the PU-OFDM from the received NOMCR signal. 

### 4.2. Sparse De-Noising Deep Auto-Encoders as Cyclostationary FRESH Equalizers

A traditional auto-encoder neural network [27,29,30] is an unsupervised learning algorithm that works with the help of the backpropagation algorithm. It consists of an encoder and decoder. The encoder represents a deterministic mapping denoted by $f_{\theta }$ which transforms an n-dimensional input vector x into a hidden representation denoted by **y**. The hidden representation can be expressed by

(26) $$y=f_{\theta }\left(x\right)=s\cdot \left(W\cdot x+b\right)$$

where θ={W,b} denotes the parameter set where W is the d-by-d parameter matrix and **b** is the offset vector of length ‘d’. The decoder is responsible for mapping the hidden representation **y**, caused by an encoder operation, to a reconstructed vector **z** of length d. This decoding operation can be represented mathematically by

(27) $$z=g_{\theta^{\prime }}\left(y\right)=s\cdot \left(W^{\prime }\cdot y+b^{\prime }\right)$$

In the traditional auto-encoder, the output of the decoder is not an exact representation of the input vector. Rather, it can be represented in probabilistic terms as the parameters of a distribution P(X|Z=z) that generates x with high probability. As pointed out in Reference [27], the decoding operation involves optimization of a reconstruction error with respect to a loss function $L\left(x,z\right)≅{\left|\left|x-z\right|\right|}^{2}$. 

The deep de-noising auto-encoder (DDA) is a variant of the traditional auto-encoder, where the corrupted input signal is mapped to a hidden representation, $f_{\theta }\left(x\right)=\mathrm{sigmoid}\left(W\cdot x+b\right)$ using multiple layers of encoder and decoder. This can be achieved by joining the layers of the decoders to form a single decoder layer. The objective function in the DDA is the minimization of the squared error loss on the entire data set between the reconstructed output signal and the desired signal as mathematically expressed in Equation (28):

(28) $$E=\frac{1}{N}\sum_{i=1}^{N}{\left|\left|x_{i}-z_{i}\right|\right|}^{2}$$

**Figure 2:**
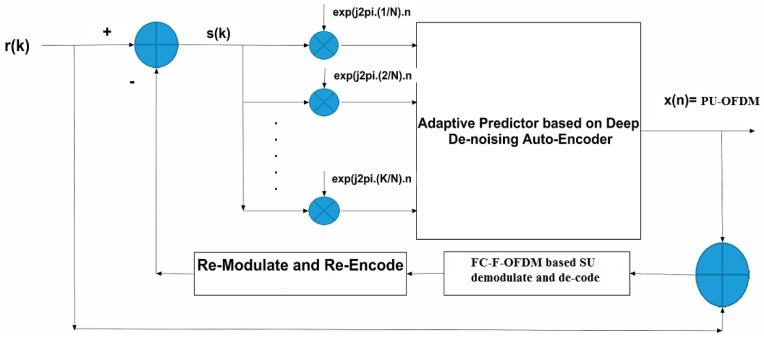
Cyclic FRESH filtering using Deep De-noising Auto-Encoder (DDA) in NOMCR signal demodulation.

The minimization of Equation (30) is done with respect to the weights using stochastic gradient descent approach. The concept of DDA can be extended to the area of cyclostationarity based adaptive interference cancellation using the concept of prediction filtering [25,26]. The initial stage of FC-F-OFDM based SU signal extraction from the NOMCR signal is implemented using the DDA as shown in Figure 2. 

The DDA based FRESH filtering steps are tabulated below (Algorithm 2).

As depicted in Figure 2, the error signal between the extracted PU-OFDM and the received NOMCR signal contains the FC-F-OFDM based SU signal. This is used for consecutive demodulation and channel decoding, as well as re-encoding and re-modulation of the estimated bit-stream of the PU-OFDM. The latter is fed back for cancellation from the received composite signal. The feedback and subsequent cancellation of estimated PU-OFDM from the received NOMCR, increases the correlation between the residual signal and its cyclic shifted versions, leading to improve desired signal estimation. As a consequence, the accuracy of estimation of the SU component in the NOMCR signal is improved. The details of auto-encoder operation are described in [27,29,30,31]. Here, we provide the outline of the backpropagation-based auto-encoder algorithm for component layer extraction from the received signal. 

From the discussion above, it is evident that some similarities and dissimilarities exist between the DDA-FRESH and RLS-FRESH structures. These are tabulated below (Table 1).
**Algorithm 2.** DDA based Cyclostationary FRESH filtering steps.**Parameters**: W = {$W^{\left(1\right)},$
$W^{\left(2\right)}$} = weights of encoding and decoding layers, b = {$b_{x},$
$b_{h}$} = biases of encoding      and decoding layers, $w_{ij}^{l}$ = connectiong weight between *j*th neuron in layer (l-1) and *i*th neuron in       layer (l), β = sparsity penalty term, $\alpha_{1}$ and $\alpha_{2}$ are L_1_ and L_2_ non-negativity constraint weight      penalty factors, ξ = learning rate of the auto-encoderInitialization:-        m = number of training samples        $s_{l}$ = number of layers in the neural network        $x_{input}\left(n\right)={\left[s\left(n\right)\cdot e^{j2\pi \vartheta_{1}n},\;s\left(n\right)\cdot e^{j2\pi \vartheta_{2}n},\ldots ,s\left(n\right)\cdot e^{j2\pi \vartheta_{K}n}\right]}^{T}$        **r**(n) = desired signal i.e., the received NOMCR signal Computation:-  Minimize the cost function, $J_{DDA}\left(W,b\right)=J_{AE}+\beta \cdot \sum_{r=1}^{n^{\prime }}D_{KL}(p||\frac{1}{m}\sum_{k=1}^{m}h_{r}\left(x_{input}^{\left(k\right)}\right))+\sum_{l=1}^{2}\sum_{i=1}^{s_{l}}\sum_{j=1}^{s_{l+1}}f\left(w_{ij}^{l}\right)$
               where    $J_{AE}=\frac{1}{m}\sum_{k=1}^{m}\;||\sigma (W^{\left(2\right)}\sigma (W^{\left(1\right)}x_{input}^{\left(k\right)}+b_{x})+b_{h})-r^{k}|{|}_{2}^{2}$
                         $h_{j}\left(x_{input}^{\left(k\right)}\right)=\sigma \left(W^{\left(1\right)}x_{input}^{\left(k\right)}+b_{x,j}\right)$, where $\sigma \left(x_{input}^{\left(k\right)}\right)=\tanh(x_{input}^{\left(k\right)})$
                         $f\left(w_{ij}\right)=\alpha_{1}\Gamma \left(w_{ij},\kappa \right)+\frac{\alpha_{2}}{2}{\left|\left|w_{ij}\right|\right|}^{2},\;w_{ij}<0$
                         And $f\left(w_{ij}\right)=0,w_{ij}\geq 0\;$
  Weight Update:        $w_{ij}^{l}=w_{ij}^{l}-\xi \cdot \frac{\delta }{\delta w_{ij}^{\left(l\right)}}J_{DDA}\left(W,b\right)$
   Bias Update:           $b_{i}^{\left(l\right)}=b_{i}^{\left(l\right)}-\xi \cdot \frac{\delta }{\delta b_{i}^{\left(l\right)}}J_{DDA}\left(W,b\right)$
   Partial derivative:       $\frac{\delta }{\delta w_{ij}^{\left(l\right)}}J_{DDA}\left(W,b\right)=\frac{\delta }{\delta w_{ij}^{\left(l\right)}}J_{AE}\left(W,b\right)+\beta \cdot \frac{\delta }{\delta w_{ij}^{\left(l\right)}}D_{KL}(p||\frac{1}{m}\sum_{k=1}^{m}h_{r}\left(x_{input}^{\left(k\right)}\right))+g\left(w_{ij}^{l}\right)$
                         $g\left(w_{ij}^{l}\right)=\alpha_{1}\cdot \nabla_{w}\left|\left|w_{ij}\right|\right|+\alpha_{2}\cdot w_{ij},\;for\;w_{ij}<0$
                           $\;\;\;\;\;=0,\;\;\;\;\;\;\;\;\;\;\;\;for\;w_{ij}>0$


**Figure 3:**
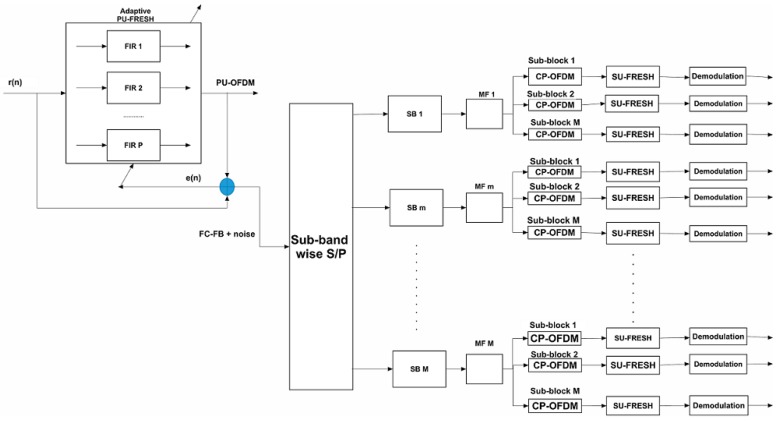
Cyclic FRESH filtering based SIC decoding in proposed NOMCR system.

The general block diagram depicting the overall interference cancellation and estimation procedure is presented in Figure 3. In this iterative scheme, the PU-OFDM signal can be treated as cyclostationary noise, which is based by a FRESH filter based on knowledge of its cyclic period. An initial estimate of the SU-FC-FB can be obtained by subtracting the estimated noisy PU-OFDM from the received mixture. As evident, the 1st adaptive FRESH filter operating based on PU cyclic parameters can be either RLS based FRESH or DDA based FRESH filter. The initial estimate is passed through a serial-to-parallel (S/P) converter which divides the incoming signal into parallel sub-channels. Each sub-channel corresponds to each sub-band of the transmitted FC-FB. There is M such sub-bands. Each sub-channel is operated by a block diagonal matrix with R AFB transform matrices on the main diagonal.

Each AFB transform matrix corresponds to each sub-block of that sub-band. Therefore, the AFB sub-matrices act as match filters (MFs) to recover the CP-OFDM based sub-blocks. Then, FRESH filter, adapted to each sub-block CP-OFDM’s cyclic frequency, can jointly act as a channel equalizer and noise canceller. The FRESH based equalized sub-block symbols are then demodulated to obtain an estimate of the transmitted bit-stream.

## 5. Results

### 5.1. Simulation Setup

The following table lists the parameters used in the computer simulation of the system (Table 2).

In this work, the PU signal is a 4-QAM modulated OFDM signal with rectangular pulse shaping filter. The FFT size for the PU-OFDM is chosen to be 32 samples, with corresponding CP size 8 samples. The same parameters for the SU-FC-FB signal are 128 and 32 respectively. It is assumed that the SU transmitter performs spectrum sensing before scheduling its transmission. It superimposes its transmission on consecutive PU-bands of size 32 FFT bins, where the IT threshold is not exceeded. The cyclic frequency set chosen, corresponding to the PU-OFDM signal, is {0, $\frac{3}{40}$, $\frac{-3}{40}$}, where each non-zero cyclic frequency is not a multiple of that of the SU-FC-FB signal. The cyclic frequency set chosen for the SU-FC-FB is {$\frac{\pm 3}{160},\;0,\;\frac{\pm 1}{160}\}$. 

A 2-layer DDA is selected, where the number of input neurons of the 1st auto-encoder layer is 640, while the numbers of hidden neurons in 1st and 2nd auto-encoder layers are 196 and 20 respectively. The number of iterations performed by the FRESH equalizer, for repeated interference estimation and subtraction, is 3. Out of 10000 samples used, the first 2000 samples are used as online training data for the DDA-FRESH system. The remaining samples are used for the interference estimation and separation. 2 injection levels, −5dB and −10 dB, are adopted in this work. This means that the SU-FC-FB signal is 5 dB and 10 dB lower in power than the PU-OFDM signal. 

### 5.2. Overall System BER Improvement

**Figure 4:**
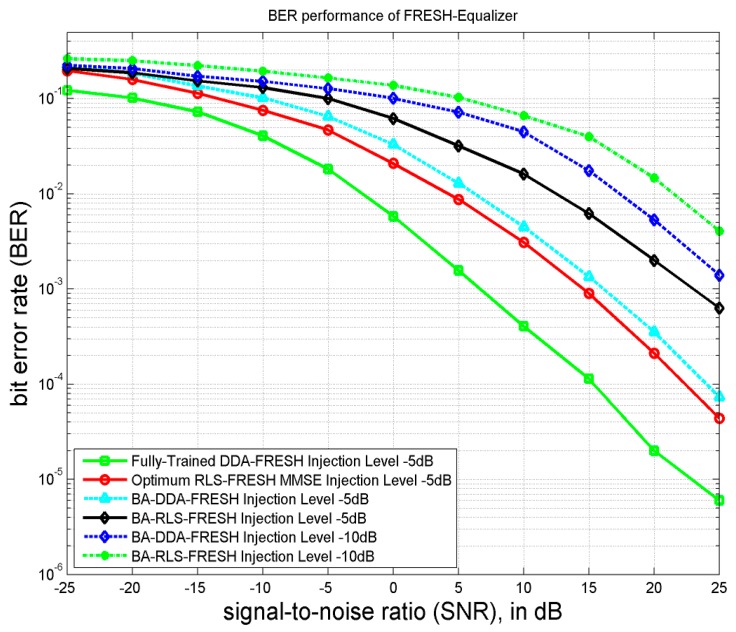
BER performance comparison between RLS based and DDA based Cyclic FRESH Equalizers for injection levels −5 dB and −10 dB.

As evident from Figure 4, the use of DDA-FRESH equalizer enhances the BER performance of the NOMCR system as compared to RLS-FRESH scheme. This is attributed to the backpropagation scheme which is involved in updating the weights and biases of the neural network. Moreover, the use of multiple layers of neurons (hidden nodes) inside the auto-encoder leads to enhanced background noise cancellation, which improves the cyclostationarity based correlation maximization operation of the FRESH filter structure. It is observed from Figure 4 that for injection level −10 dB and SNR higher than 5 dB, the BA-DDA-FRESH equalizer out-performs the BA-RLS-FRESH by 5 dB. But for a lower injection level (−5 dB) and SNR higher than 5 dB, the BA-DDA-FRESH outperforms the BA-RLS-FRESH by almost 10 dB. As a matter of fact, the performance of the BA-DDA-FRESH is very close to the optimum RLS-FRESH equalizer for an injection level of −5 dB. This improvement in performance of the BA-DDA FRESH can be attributed to the higher non-linearity cancellation via iterative interference estimation and the subtraction procedure adopted. 

### 5.3. System SINR Improvement at −5 dB Injection Level

**Figure 5:**
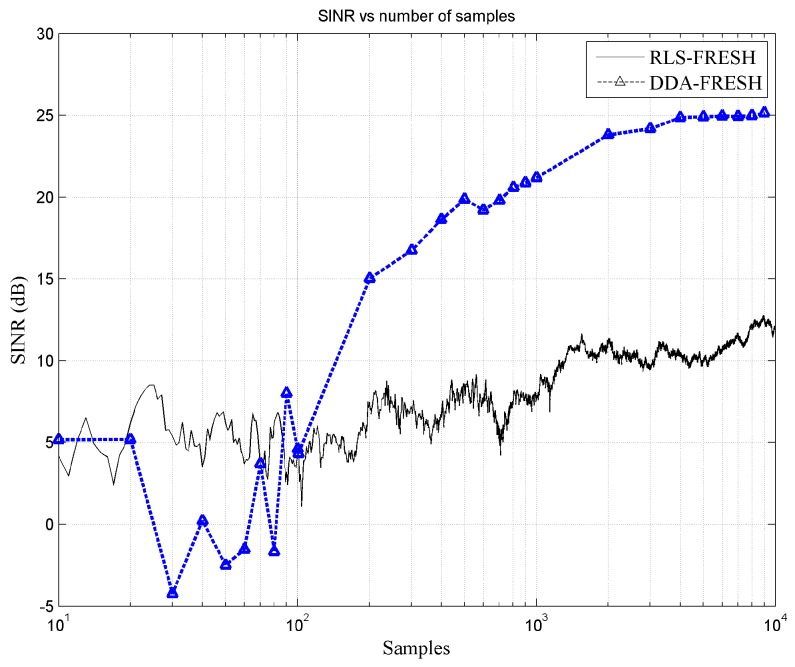
SINR vs total number of samples at SNR 5 dB and injection level −5 dB, demonstrating comparative performance of RLS-FRESH and DDA-FRESH equalizers with regards to successive interference cancellation based NOMCR decoding.

In Figure 5, the SINR performance improvement of DDA-FRESH over RLS-FRESH, for the first 10,000 samples, is presented at SNR 5 dB and injection level −5 dB. Clearly from Figure 5, we can see that the decoding operation of the NOMCR scheme using the auto-encoder based FRESH equalizer outperforms the conventional RLS-FRESH scheme. At SNR 5 dB and injection level −5 dB, the performance of the DDA-FRESH significantly improves after 100 samples. This is attributed to the enhancement in cyclostationarity recognition performance due to iterative interference estimation and successive cancellation procedure. 

**Figure 6:**
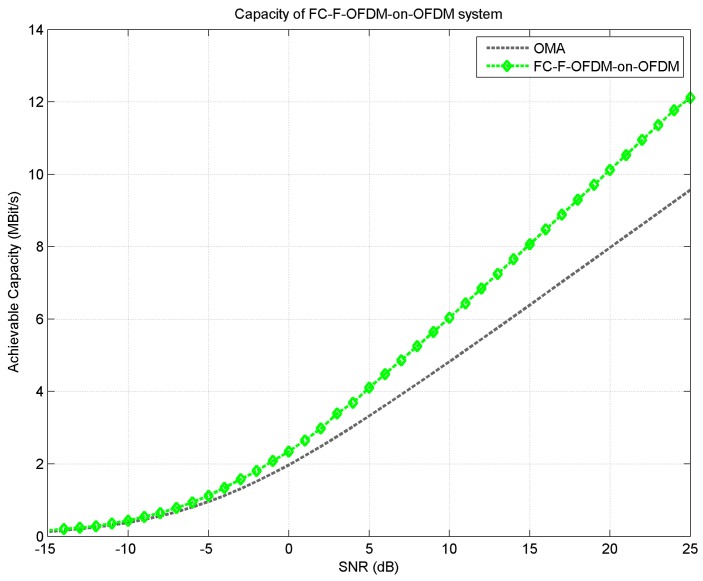
Achievable capacity comparison between conventional OMA and proposed FC-F-OFDM-on-OFDM based LDM system.

The achievable capacity advantage of LDM based NOMCR system over conventional orthogonal multiple access (OMA) based cognitive transmission is presented in Figure 6 above. As evident, the use of NOMCR scheme clearly outperforms the conventional cognitive orthogonal access scheme.

## 6. Future Applications

The above described FRESH-SIC receiver approach can be applied in a variety of areas such as Fog-Radio Access Networks (F-RANs) and Heterogeneous NOMA networks. Some of the works in these application areas are presented in References [32,33]. 

### 6.1. F-RAN Scenario

In Reference [32], the authors present a system level architecture of NOMA-based F-RAN. In that work, multiple F-RAN systems co-exist within a macro-cell. In such an architecture, NOMA can be implemented between the F-UEs served by the same Fog Access Point (F-AP), as well as between different F-APs which share the same sub-channel. In this type of multi-layered architecture, a group of F-UEs in device-to-device (D2D) mode can transmit various contents like audio, video and text through multiple bandwidth channels. Evidently, channel conditions are different among the different F-UEs. The received signal at any F-UE includes the desired signal, AWGN, cross-tier interference from the M-RRH, co-tier interference (i.e., from F-UEs served by other F-AP) as well as interference from NOMA based F-UEs served by the same F-AP. In this work, the authors formulate the power and sub-channel allocation in a F-RAN system as a sum rate maximization problem, from a game-theoretic point of view. Similarly to the underlay cognitive NOMA concept, the authors in Reference [32] propose to protect the Macro Remote Radio Head (M-RRH) by setting a maximum tolerable interference temperature (IT) threshold. Moreover, it is proposed to use SIC to remove co-tier interference. Their proposition is to constrain the maximum number of Fog User Equipments (F-UEs) occupying the same sub-channel at the same time, in order to reduce the decoder complexity. However, in such an environment where each F-UE encounters both co-tier and cross-tier interference, the FRESH-SIC scheme described in our work might prove beneficial. By taking into account the modulation format and the signal repetition patterns of the individual component signals, the proposed neuromorphic FRESH-SIC receiver can be incorporated in an F-RAN scenario to provide better BER performance and SINR improvements in a low SNR regime. 

### 6.2. NOMA Based Heterogeneous Network Model

The authors in Reference [33] have proposed energy efficient resource allocation in a NOMA based heterogeneous network. In their work, they describe an overlay system architecture in which a macro-cell with a macro base station (macro-BS) is overlaid with multiple small cells, each small cell incorporating the power domain multiplexing-based NOMA technique. In such a system, multiple small cell-UEs (SUEs), operating within each small cell, are multiplexed on the same sub-band. It is also possible to co-multiplex Macro-cell UEs (MUEs) with the SUEs on the same sub-band. The energy efficiency in such a system is defined to be the ratio of total system sum-rate to the total power consumption. The work focusses on energy efficiency maximization by formulating a probabilistic mixed non-convex optimization problem, under the constraints of outage probability, maximum transmission power and total number of multiplexed users on each sub-band. In this work also, the authors apply conventional SIC for NOMA decoding. However, under low SNR conditions, a conventional SIC based receiver can impair system performance. A receiver architecture, which can operate under very low SNR conditions, can relax the constraints on maximum number of users multiplexed within a sub-band. The proposed FRESH-SIC structure can be applicable in such a scenario for further analysis.

## 7. Discussion

In this work, a neuromorphic cyclostationary receiver is proposed for a non-orthogonal multiplexed cognitive radio system (NOMCR). The system operates on the principle of a non-orthogonal layer division multiplexed (LDM) system. Preliminary computer simulations have been carried out in this work, which indicate the potential of neural network-based equalization in such an environment. In this work, a de-noising auto-encoder based on sparsity principle has been used to operate as a cyclostationary prediction filter-based equalizer. The result shows that it outperforms the conventional cyclostationarity based equalizer. Moreover, the use of fast convolution-based filter-bank processing has been proposed for the multicarrier NOMA scheme. In future works, transmitter side PU interference avoidance schemes can be designed leading to channelized NOMA architectures for better PU protection as well as higher spectral efficiency. Moreover, advanced neuromorphic receiver design based on combined cyclostationarity processing and deeper neural architectures can be considered as well.

## Figures and Tables

**Table 1:** Comparative Features between RLS-FRESH and DDA-FRESH.

Features	RLS-FRESH	DDA-FRESH
Adaptive	Yes	Yes
De-noising Ability	Yes	Yes
Tracking Ability	Yes	Yes
Non-linear	No	Yes
Inter-connectivity	Limited	Massive
Architecture	Single Layer Parallel Filter-bank	Multiple Cascaded Layer
Error Propagation	Feed-back	Back-propagation
Convergence Speed	Faster	Slower
Blind Adaptive Capability	Little or No training data required	Certain amount of training data required
Prediction Performance	Good, under Gaussian noise	Better than RLS-FRESH, under non-Gaussian noise

**Table 2:** Simulation Parameters.

SU Symbol Level Modulation Type	16-QAM
PU Symbol Level Modulation Type	4-QAM
SU pulse shaping filter	Root Raised Cosine (RRC)
PU pulse shaping filter	Rectangular
SU Injection level	5 dB, 10 dB
Total frame length of PU-OFDM (without CP)	32 samples
CP length of PU-OFDM	8 samples
Total sub-block length of SU-OFDM (without CP)	128 samples
CP length of SU-OFDM sub-block	32 samples
Total number of samples used	10000
Number of hidden layer nodes in 1st layer	196
Number of hidden layer nodes in 2nd layer	20
Sparsity Parameter	0.05
Weight decay parameter	0.003
Sparsity Penalty term Weight	3
